# Acute venous thromboembolism plasma and red blood cell metabolomic profiling reveals potential new early diagnostic biomarkers: observational clinical study

**DOI:** 10.1186/s12967-024-04883-8

**Published:** 2024-02-24

**Authors:** Cláudia Febra, Joana Saraiva, Fátima Vaz, João Macedo, Hamza Mohammad Al-Hroub, Mohammad Harb Semreen, Rui Maio, Vitor Gil, Nelson Soares, Deborah Penque

**Affiliations:** 1 Department of Intensive Care, Hospital da Luz Lisboa, Lisbon, Portugal; 2 Faculty of Medicine, University of Porto, Porto, Portugal; 3 Human Genetics Department, Instituto Nacional de Saúde Dr. Ricardo Jorge (INSA), Lisbon, Portugal; 4 Center for Toxicogenomics and Human Health (ToxOmics), NOVA Medical School-FCM, UNL, Lisbon, Portugal; 5 NOVA School of Science and Technology, Universidade Nova de Lisboa, Lisbon, Portugal; 6 Department of Medical Chemistry, College of Pharmacy, University of Sharjah, Sharjah, United Arab Emirates; 7 Sharjah Institute for Medical Research, University of Sharjah, Sharjah, United Arab Emirates; 8 Department of General Surgery, Hospital da Luz Lisboa, Lisbon, Portugal; 9 Center of Cardiovascular Risk and Thrombosis, Hospital da Luz Torres de Lisboa, Lisbon, Portugal

**Keywords:** Pulmonary embolism, Deep vein thrombosis, Venous thromboembolism, Metabolomics

## Abstract

**Background:**

Venous thromboembolism (VTE) is a leading cause of cardiovascular mortality. The diagnosis of acute VTE is based on complex imaging exams due to the lack of biomarkers. Recent multi-omics based research has contributed to the development of novel biomarkers in cardiovascular diseases. Our aim was to determine whether patients with acute VTE have differences in the metabolomic profile compared to non-acute VTE.

**Methods:**

This observational trial included 62 patients with clinical suspicion of acute deep vein thrombosis or pulmonary embolism, admitted to the emergency room. There were 50 patients diagnosed with acute VTE and 12 with non-acute VTE conditions and no significant differences were found between the two groups for clinical and demographic characteristics. Metabolomics assays identified and quantified a final number of 91 metabolites in plasma and 55 metabolites in red blood cells (RBCs). Plasma from acute VTE patients expressed tendency to a specific metabolomic signature, with univariate analyses revealing 23 significantly different molecules between acute VTE patients and controls (*p* < 0.05). The most relevant metabolic pathway with the strongest impact on the acute VTE phenotype was d-glutamine and d-glutamate (*p* = 0.001, false discovery rate = 0.06). RBCs revealed a specific metabolomic signature in patients with a confirmed diagnosis of DVT or PE that distinguished them from other acutely diseased patients, represented by 20 significantly higher metabolites and four lower metabolites. Three of those metabolites revealed high performant ROC curves, including adenosine 3′,5′-diphosphate (AUC 0.983), glutathione (AUC 0.923), and adenine (AUC 0.91). Overall, the metabolic pathway most impacting to the differences observed in the RBCs was the purine metabolism (*p* = 0.000354, false discovery rate = 0.68).

**Conclusions:**

Our findings show that metabolite differences exist between acute VTE and nonacute VTE patients admitted to the ER in the early phases. Three potential biomarkers obtained from RBCs showed high performance for acute VTE diagnosis. Further studies should investigate accessible laboratory methods for the future daily practice usefulness of these metabolites for the early diagnosis of acute VTE in the ER.

**Supplementary Information:**

The online version contains supplementary material available at 10.1186/s12967-024-04883-8.

## Background

Venous thromboembolism (VTE) is a leading cardiovascular disease in terms of both incidence and mortality, and ranks among the top three [1]. Much less investigated than arterial thrombosis, VTE is a complex multifactorial entity manifested by a spectrum of disease that ranges from pulmonary embolism (PE), as its most severe manifestation, to deep vein thrombosis (DVT), as the most common presentation [2]. Survival after VTE is heavily reduced, both short and long term, with 6% of patients not surviving the first 30 days after the acute event [3]. Acute VTE events may be further complicated by chronic thromboembolic pulmonary hypertension in 3% of PE cases, whereas 20–50% of DVT patients develop chronic post-thrombotic syndrome [4]. The diagnosis of VTE is only possible after a considerable workup, due to the high heterogeneity of symptoms [5, 6]. The absence of available diagnostic biomarkers necessitates an approach in the emergency setting based on clinical risk factors to determine the indication for gold-standard imaging exams, such as thoracic CT angiography and lower limb Doppler ultrasound. These imaging modalities are essential for a definitive diagnosis, but they present several challenges, including accessibility issues worldwide, high resource expenditure, long wait times for results, and unsafe radiation exposure [7]. The use of a single biomarker, or a panel, to distinguish patients with acute VTE is critical to modify the negative outcomes still associated.

Multiomics analysis of venous thrombosis, propelled by the use of contemporary high-throughput approaches, contributed with recent data on possible biological processes involved in VTE [8]. Acute PE plasma metabolomics revealed significant differences in metabolites associated with energy imbalance and signal transduction mediators [9]. Concomitantly, DVT fresh thrombus metabolic profiling detected high levels of metabolites involved in glycolysis, purine and tryptophan metabolism, and redox reactions [10]. The search for new biomarkers for early and accurate VTE diagnosis is a challenge and with scarce data available, but we know from studies in other areas that easily accessible biologic samples are considered as the most interesting carriers of the new biomarkers [11]. On the other hand, biomarkers in serum or urine are usually present at low concentrations, depending on advances in highly sensitive analytical techniques to its identification and quantification.

In this context, we decided to compare the metabolic profile of plasma and RBCs obtained from patients admitted to an emergency room (ER) for acute VTE with those of non-acute VTE patients with the same presenting symptoms. Our aim was to identify metabolites differentially associated with acute VTE during the early stages of the disease that could integrate a potential future panel of diagnostic biomarkers.

## Methods

### Study population

**Fig. 1:**
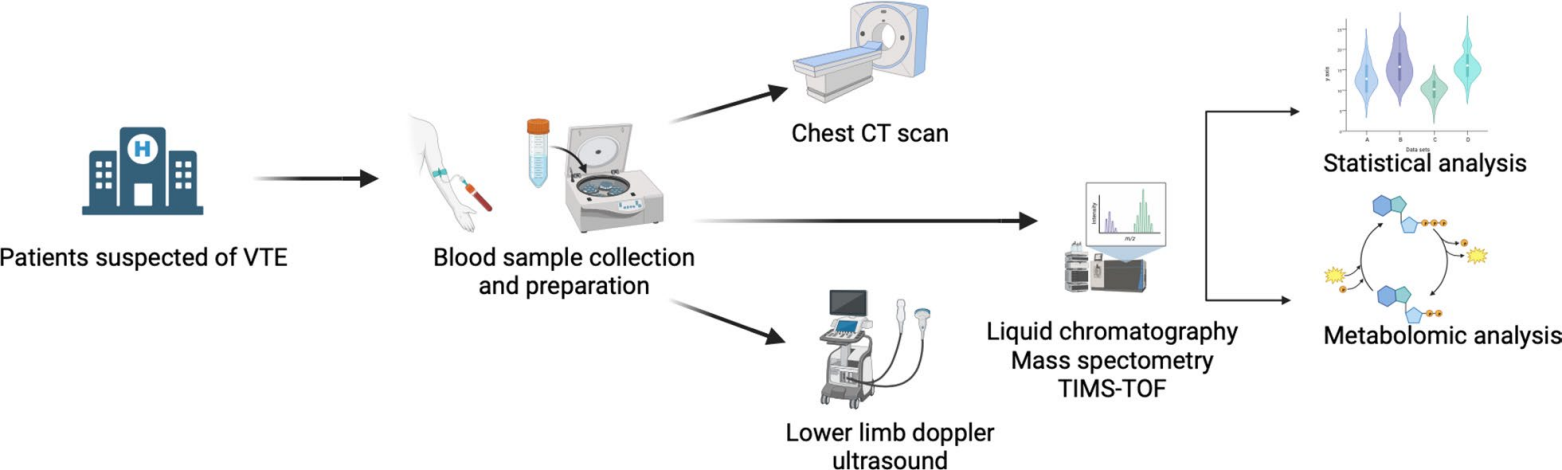
Study workflow

Consecutive adult patients (n = 62, ≥ 18 years), attending the ER of Hospital Beatriz Angelo, Lisbon, between 8 a.m. and 1 p.m., with symptoms/signs of suspected acute PE or acute DVT and without any diagnosed active cancer, pregnancy, or recent prolonged immobilization and/or surgery, were recruited for this study (Fig. 1). The suspicion of VTE was clinically established by the attending physician. All patients were submitted to definite diagnostic imaging, chest CT angiography for PE suspicion and lower limb Doppler ultrasound for DVT, according to usual practice. All patients were discharged or transferred to another department with an established final diagnosis. Demographic and clinical data referring to the first 24 h were recorded. Ethics Review Board of Hospital Beatriz Ângelo approved the study with the number 0421 (2020) and this ethical approval was recognised by the Ethical Commission of National Institute of Health Dr Ricardo Jorge (INSA). The samples will be stored until 20 years after collection. All patients included in this study gave informed consent for the purposes of research.

### Samples from the Biobank

#### Sample preparation

Every sample obtained was processed until a maximum of four hours after collection and submitted to the same sample treatment protocol. In short, 5 mL of whole blood was collected using K2EDTA tubes, which were centrifuged at 3000 rpm for 10 min at 4 °C. The plasma was separated from whole blood and immediately stored at optimal conditions. After removal of the buffy coat (saved for later analysis), RBCs were washed with 1 × PBS using centrifugation at 1800 rpm for 5 min at 4 °C. The supernatant was discarded, and the wash was repeated one more time. The resulting aliquots of plasma, RBCs and white blood cells were all stored at -80 °C. These samples were regularly transported to the biobank, taking a maximum of 30 min during transportation, and were moved inside dry ice containers.

### Metabolomics assays

#### Preparation of RBC samples

The samples were prepared as described by Gehrle et al. [12]. Briefly, 10 µl of RBCs was added to 90 μL of cold methanol/acetonitrile/water (5:3:2- v/v/v). The mixture was shaken at 700 rpm for 30 min at 4 °C and immediately centrifuged at 18,213 g and 10 min, 4 °C. The upper phase was collected and evaporated by speed vacuum.

#### Preparation of plasma samples

Metabolites were extracted from plasma (10 μL) using 90 μL of 74.9:24.9:0.2 (vol/vol/vol) acetonitrile/methanol/formic acid as reported by Hernández-Alonso P et al. [13]. The samples were vortexed at 1 min, 4 °C and centrifuged at 10min, 18,213 g, 4 °C. The upper phase was collected and evaporated by speed vacuum.

### Metabolite extraction and sample preparation

Plasma or RBC samples were resuspended at room temperature, and 100 μL of the samples from each group was mixed with 300 μL methanol (≥ 99.9%, LC‒MS CHROMASOLV) for protein precipitation, vortexed and incubated at − 20 °C for 2 h. The samples were vortexed again and centrifuged at 14,000 rpm for 15 min, and the supernatants were transferred to new glass vials. The supernatants were evaporated in a SpeedVac EZ-2 Plus (GeneVac, Ipswich, UK) at 35–40 °C. To prepare the extracts for liquid chromatography coupled to mass spectrometry (LC‒MS) analysis, they were first resuspended in 200 µl of deionized water containing 0.1% formic acid (LC‒MS CHROMASOLV, Honeywell, Seelze, Germany) and vortexed for 2 min for complete mixing. The extracts were then filtered using a 0.45 µm hydrophilic nylon syringe filter for LC‒MS/MS analysis. Equal volumes (10 µl) of 30 samples from each study group were collected and pooled for quality control (QC) and placed in the autosampler at 4 °C to analyse the reproducibility of the analysis.

### Ultrahigh-performance liquid chromatography coupled with electrospray ionization and quadrupole time-of-flight mass spectrometry (UHPLC-ESI-QTOF-MS)

An ultrahigh-performance liquid chromatography system (UHPLC) (Bruker Daltonik GmbH, Bremen, Germany) was used in conjunction with a quadrupole time-of-flight mass spectrometer for the LC‒MS/MS analysis (TIMS-QTOF). The system was equipped with an electrospray ionization (ESI) source, a solvent delivery systems pump (Elute UHPLC HPG 1300), an autosampler, and a thermostat column compartment. Windows 10 Enterprise 2016 LTSB was used as the computer operating system. The computer operating system was Windows 10 Enterprise 2016 LTSB, and the software used was Bruker Compass HyStar 5.0 SR1 Patch1 (5.0.37.1), Compass 4.1, Version 6.2. Two different mobile phases were utilized: one with water and 0.1% formic acid (A) and the other with acetonitrile and 0.1% formic acid (B). The gradient program was as follows: 0–2 min, 99% A: 1% B; 2–17 min, 99–1% A: 1–99% B; 17–20 min, 99% B: 1% A, and the flow rate was fixed at 0.25 mL/min. Subsequently, 20–20.1 min 99% B to 99% A; 20.1–28.5 min, 99% A: 1% B at 0.35 mL/min flow rate; 28.5–30 min; 99% A: 1% B at 0.25 mL/min. A Hamilton^®^ Intensity Solo 2 C18 column (100 mm × 2.1 mm × 1.8 m) was employed for separation. The oven temperature was maintained at 35 °C. The drying gas flow rate was 10.0 L/min (220 °C), the capillary voltage was 4500 V, and the nebulizer pressure was 2.2 bar in the ESI. The collision energy ranged from 100 to 250% and was set at 20 eV with a 500 V end plate offset for MS2 acquisition. For the external calibration, sodium formate was used. For the calibrant sodium formate, the auto MS scan segment used for acquisition ranged from 0 to 0.3 min, and for auto MS/MS, it ranged from 0.3 to 30 min. The acquisition was performed in positive mode at 12 Hz in both segments, and the automatic in-run mass scan range was 50–1300 m/z. With a cycle length of 0.5 s and a threshold of 400 cts, the width of the precursor ion was 0.5. After three spectra, the active exclusion was removed and released after 0.2 min. Mass calibration was performed prior to analysis according to the manufacturer’s recommendations using external mass calibration (10 mM sodium formate calibrant solution). TRX-2101/RT-28-calibrants for Bruker T-ReX LC-QTOF (Nova Medical Testing Inc.) were injected before sample analysis to check and test the performance of the column, reversed-phase liquid chromatography (RPLC) separation, multipoint retention time calibration, and the mass spectrometer. Additionally, TRX-3112-R/MS Certified Human serum for Bruker T-ReX LC-QTOF solution (Nova Medical Testing Inc.) was prepared from pooled human blood and injected before sample analysis to check the performance of the LC‒MS instruments. The analysis was performed using a randomized sequence order with five injections of solvent A (0.1% formic acid in deionized water) sample at the beginning of the sequence for apparatus equilibration, followed by five injections of the pooled QC sample. Additionally, one QC injection was performed every (9–10 samples) to minimize the carry-over and evaluate the reproducibility of the analysis and monitor batch effects [14].

### Data processing and analysis

The MS data (Bruker Daltonik GmbH, Bremen, Germany) were preprocessed through the MetaboScape^®^ 4.0 program. The T-ReX 2D/3D approach employed the peak area to quantify the feature after bucketing the processed data with an intensity threshold of 1000 and a peak length of 7 spectra. The calibration for mass spectra was performed in 0–0.3 min, and chromatographic peak widths less than 0.03 min were not included in the analysis. The auto MS/MS scan was performed using the average method, with scan parameters ranging from 0.3 to 25 min of retention time and 20 to 1300 m/z of mass. LC‒MSMS-QTOF was used to examine all of the study samples in duplicate. Unreliable features were removed using the QC samples. Human Metabolome Database (HMBD) 5.0 metabolomics database was used to map the MS/MS spectra and retention time to identify the metabolites. The selected metabolites were filtered by picking a higher annotation quality score that indicates the MS/MS score, m/z values, best retention time, mSigma, and analyte list spectrum library, and the annotation procedure was followed to identify the compounds with MS/MS using library matching. The peak intensity of each metabolite obtained was used for quantification of the data matrix, and only significant compounds with a *p* value of < 0.05 were selected from HMDB 5.0.

### Statistical analysis

Clinical and demographic variables were described using absolute and relative frequencies for categorical variables and median and interquartile ranges (IQR) for continuous variables. Comparisons between confirmed acute VTE patients and control patients were performed by means of one multifunctional metabolomics data analysis platform with univariate and multivariate tools. The preanalytical filter procedure included the exclusion of exogenous contaminants (p.e., paracetamol, caffeine, L-DOPA, metoprolol, and buffer solution) and metabolites quantified in less than one-third of the samples. We used the metabolomics analysis protocol MetaboAnalyst 5.0 [15, 16]. After checking for normality of the distribution of untargeted LCMS-derived data from the RBCs and plasma samples, they were mean-centered and UV-scaled. Only variables present in at least 60% of any group and with intensity of at least 4.0 × 10^3^ were included for analysis. Principle component analysis (PCA) and hierarchical clustering were performed for unsupervised multivariate statistical [17]. We performed orthogonal partial-least squares discrimination analysis (OPLS-DA) as the supervised method to identify important variables with discriminative power. OPLS-DA models were validated based on multiple correlation coefficient (R^2^) and cross-validated R^2^ (Q^2^) in cross-validation and permutation test by applying 2000 iterations (*p* > 0.001). The significance of the biomarkers was ranked using the variable importance in projection (VIP) score (> 1) from the OPLS-DA model [18]. The loading plots and variable influence on projection (VIP) plots were inspected to differentiate the phenotypic groups. Hierarchical clustering analysis was performed based on the degree of similarity of metabolite abundance profiles and similarly patterned abundant metabolites were positioned together. The heat map and dendrogram indicated this distribution of abundance profiles. Univariate analysis consisted of fold change, and we considered a fold change > 1.5 to select differentially abundant metabolites. Pathway enrichment analysis used the Mummichog algorithm [19] in the “Functional Analysis” module in Metaboanalyst 5.0. A *p* value cut-off of 0.05 was used to consider metabolite matching. Receiver operating characteristic (ROC) curves were used to assess the performance of different metabolites in diagnosing acute VTE, and the Least Absolute Shrinkage and Selection Operator (LASSO) algorithm [20] was used to select biomarker candidates, while logistic regression was used to assess model performance based on ROC curves. In this exploratory metabolomics study, *p* values < 0.05 were considered statistically significant. All quantified metabolites that were matched by m/z were searched in HMDB library [21]. Statistical analyses were conducted using SAS 9.4 (SAS Institute Inc., Cary, NC, USA).

## Results

### Characteristics of study subjects

In total, 62 patients admitted to the ER were included in this study when initial suspicion of acute VTE was clinically determined. The clinical and demographic characteristics of the patients are detailed in Table 1.

**Table 1:** Demographic and clinical characteristics of acute VTE and control patients

	Acute VTE	Control	*p*
Age (years) n	50	12	0.108
Median [IQR]	64.5 [44.25–81.75]	72.5 [66.25–88.0]	
Female sex n (%)	32 (64.0)	7 (58.3)	0.748
Smoking n (%)	4 (8.0)	1 (8.3)	0.970
Diabetes n (%)	4 (8.0)	1 (8.3)	0.970
Hormone treatment n (%)	4 (12.5)	0	0.323
Anti-thrombotic drugs n (%)	2 (4.0)	2 (16.7)	0.109
Erythrocytes (*10^12^/L)	42	10	0.291
Median [IQR]	4.6 [3.98–4.81]	4.25 [3.76–4.71]	
Hemoglobin (g/dl) n	42	10	0.935
Median [IQR]	13.4 [12.47–14.50]	12.65 [10.92–13.95]	
RDW^a^ (%)	42	10	0.079
Median [IQR]	14.30 [13.08–15.00]	13.50 [13.08–14.12]	
Leucocytes (*10^12^/L)	42	10	0.725
Median [IQR]	10.01[7.37–11.37]	11.09 [7.85–13.92]	
Platelets (*10^9^/L) n	42	10	0.079
Median [IQR]	263 [213–355]	202 [184–245]	
D-dimers (mg/L) n	36	8	0.803
Median [IQR]	7.06 [3.84–11.04]	2.24 [1.06–14.38]	
Creatinine (mg/L) n	42	9	0.846
Median [IQR]	0.90 [0.76–1.07]	1.05 [0.85–1.99]	
CRP^b^ (mg/L) n	38	9	0.416
Median [IQR]	2.26 [0.77–4.52]	13.87 [1.39–19.50]	

^a^Red cell distribution width

^b^C-reactive protein

No significant difference between patients with confirmed acute VTE diagnosis and patients with other nonacute VTE diagnoses was observed. Red cell distribution width (RDW) and platelet hematologic parameters showed a tendency for higher values among acute VTE cases. A diagnosis of acute VTE was confirmed in 50 patients, and the remaining patients were attributed to another final diagnosis (Table 2). Acute PE, with or without concomitant DVT, was the most prevalent diagnosis and represented 68% of all acute VTEs.

**Table 2:** Diagnosis of acute VTE and control patients

	Acute VTE (n = 50)	Control (n = 12)
Pulmonary embolism	34	*na*
Deep vein thrombosis	24	*na*
Pulmonary embolism and deep vein thrombosis	12	*Na*
Erysipela	0	4
Superficial phlebitis	0	3
Sepsis	1	1
Pneumonia	0	1
Atrial fibrillation	0	1
Pericardial effusion	0	1
Anxiety	0	1
Neoplasia	1	0

*na* not applicable

Metabolomics assays identified and quantified a total of 112 metabolites in plasma and 68 metabolites in RBCs. After eliminating the contaminants in the preanalytical steps, a total of 91 were identified and quantified in the plasma assays and 55 metabolites in the RBCs (Additional file 1: Tables S1 and S2).

### Plasma metabolome

**Fig. 2:**
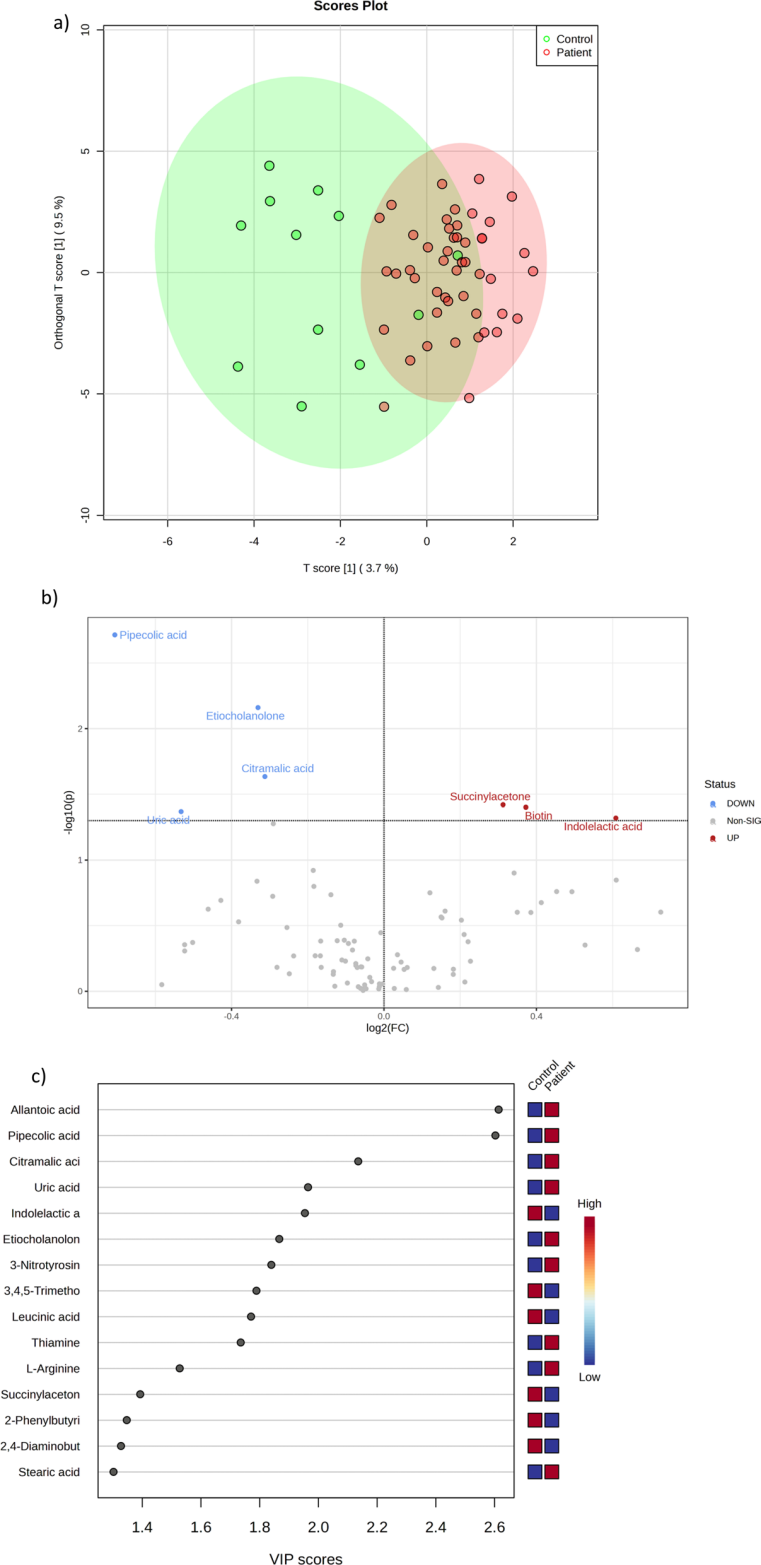

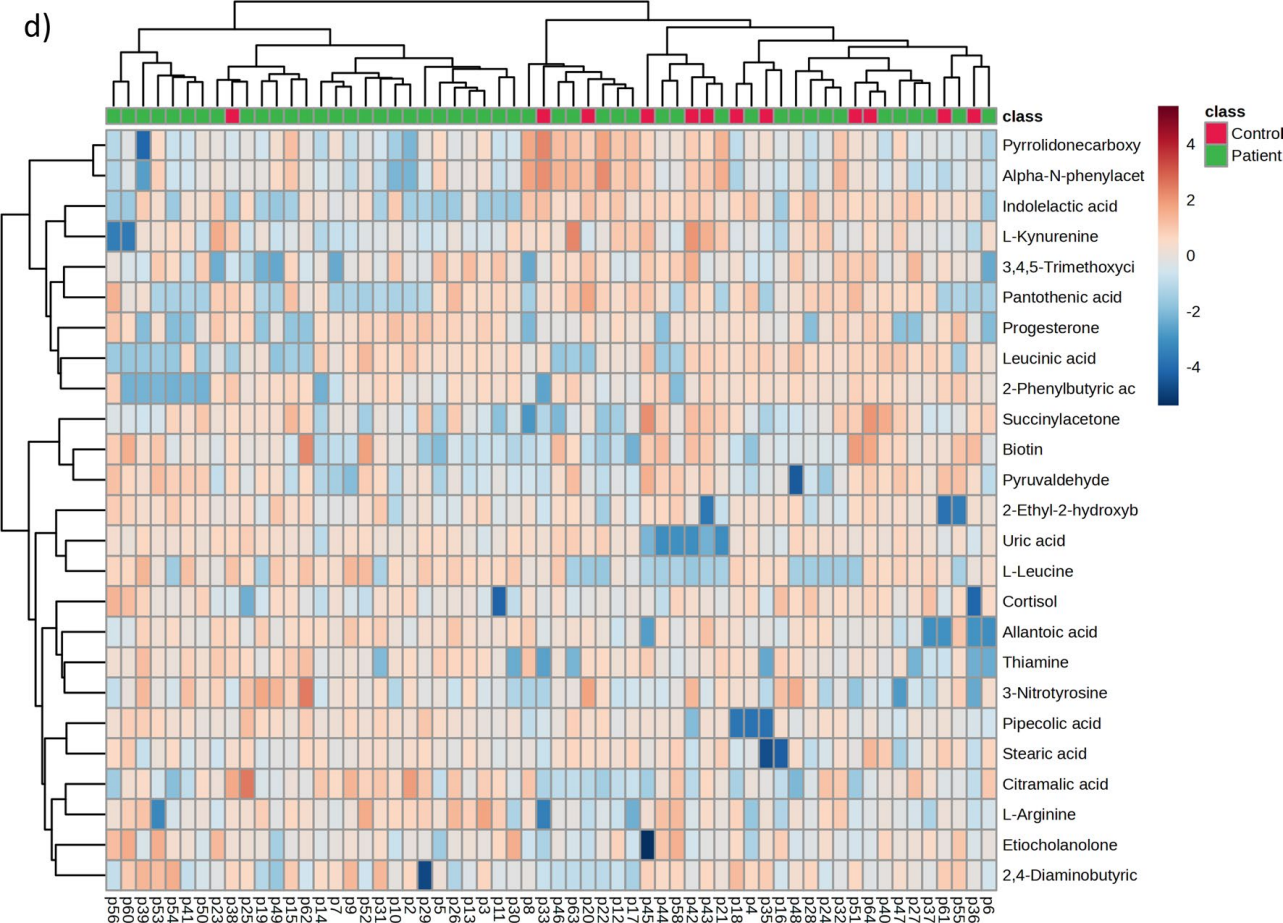
Patients with acute VTE exhibit a plasma metabolomic signature. **a** PLS-DA analyses show that the metabolome is able to discriminate between acute VTE patients and control patients, with those metabolites contributing to separation shown in **b** the volcano plot and **c** VIP score graph. **d** Heatmap hierarchical clustering analyses using the 25 metabolites with the lowest *p* value indicate that there is not perfect clustering between groups

As shown in Fig. 2, plasma from acute VTE patients expressed a tendency toward a specific metabolomic signature by both PLS-DA and hierarchical clustering analyses (Fig. 2a). The most important metabolites defining this signature (Fig. 2c, d) were allantoic acid, pipecolic acid, citramalic acid, uric acid, etiocholanolone, succinylacetone and biotin. The discrimination between acute VTE and non-acute VTE by using a hierarchical clustering algorithm (Fig. 2b) with the 25 molecules with the lowest *p* value revealed a limited performance, due to some acute VTE patients clustering together with nonacute VTE patients.

**Fig. 3:**
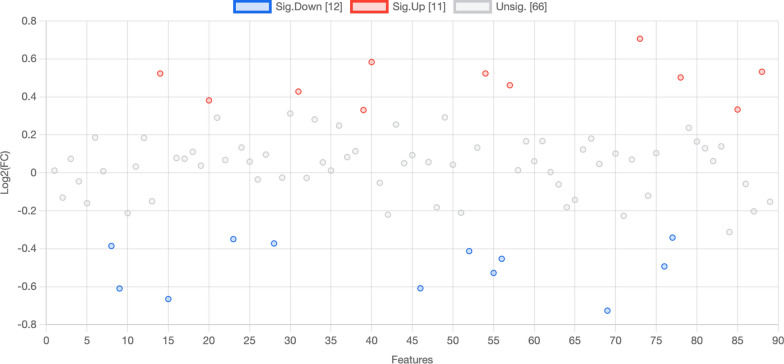
Plasma of acute VTE and control patients exhibited 23 significantly different metabolites

When applying univariate analyses, there were 23 significantly different molecules (*p* < 0.05, 11 upregulated in acute VTE patients, 12 downregulated) appeared (Fig. 3 and Table 3).

**Table 3:** Plasma metabolites significantly different between acute VTE and control patients

	log2(FC^a^)
Pantothenic acid	− 0.72629
Pipecolic acid	0.70643
4-Hydroxyproline	− 0.66
3,4,5-Trimethoxycinnamic acid	− 0.61
Indolelactic acid	− 0.61
Hippuric acid	0.58
Uric acid	0.53
L-Glutamine	− 0.53
L-Fucose	0.52
4-Aminophenol	0.52
Quinaldic acid	0.50
Pyrrolidonecarboxylic acid	− 0.49
L-Leucine	0.46
L-Kynurenine	− 0.45
Cortisol	0.43
Leucinic acid	− 0.41
2-Phenylbutyric acid	− 0.39
Adenosine monophosphate	0.38
Biotin	− 0.37
Alpha-*N*-phenylacetyl-l-glutamine	− 0.35
Pyruvaldehyde	− 0.34
Thiamine	0.33
Etiocholanolone	0.33

^a^Fold change

**Fig. 4:**
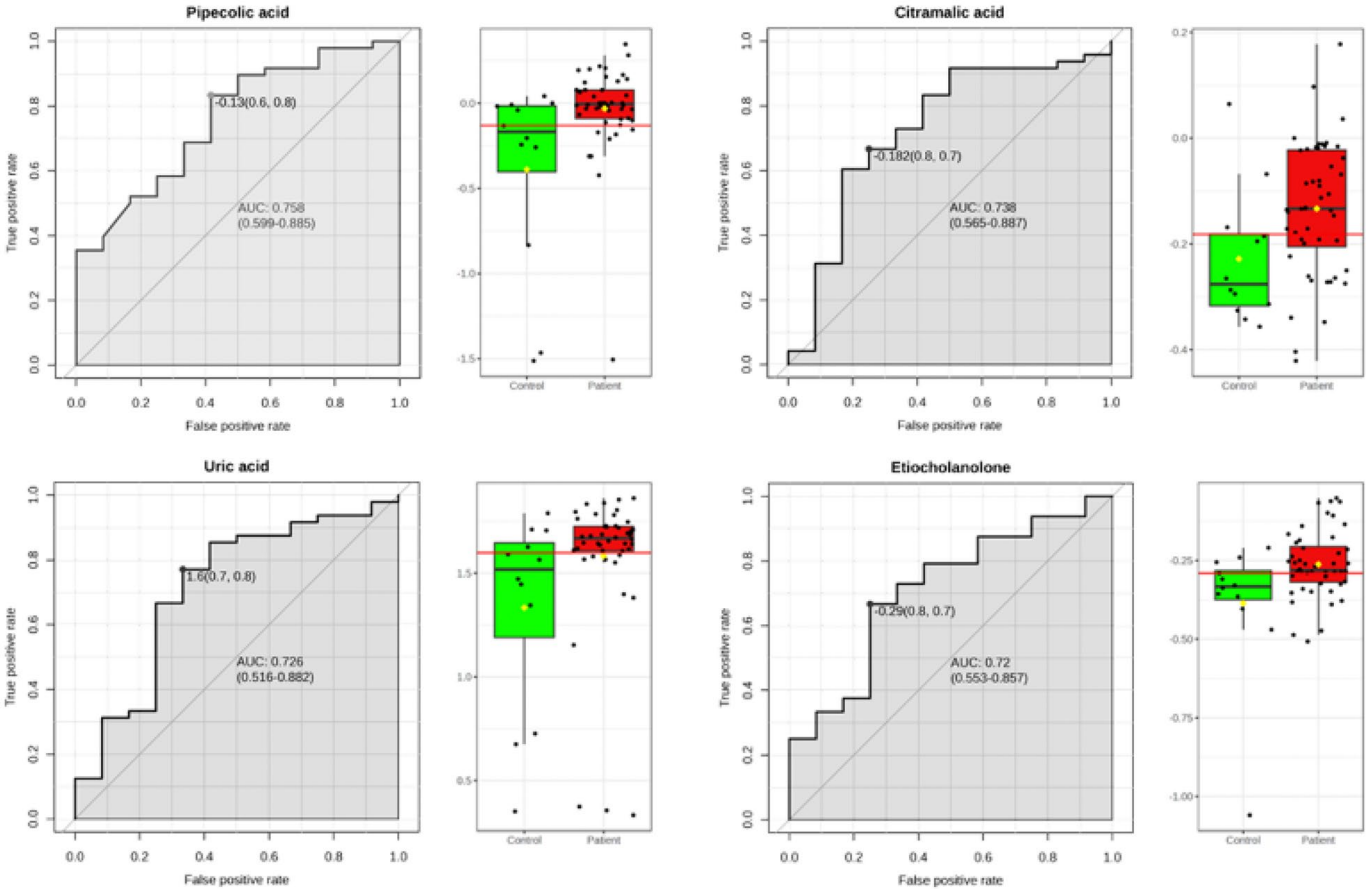
ROC curves of those plasma metabolites with higher AUC values

To define the capacity of these specific metabolites as potential biomarkers, ROC curves were generated, and we selected those with higher areas under the curve (AUCs) (Fig. 4). The best AUC were obtained by pipecolic acid (AUC: 0.758), citramalic acid (AUC: 0.738), uric acid (AUC: 0.728) and ethiocolanolone (AUC: 0.72). These ROC’s revealed that none of the metabolites had high (AUC > 0.9) por very good (AUC: 0.8–0.9) performance.

**Fig. 5:**
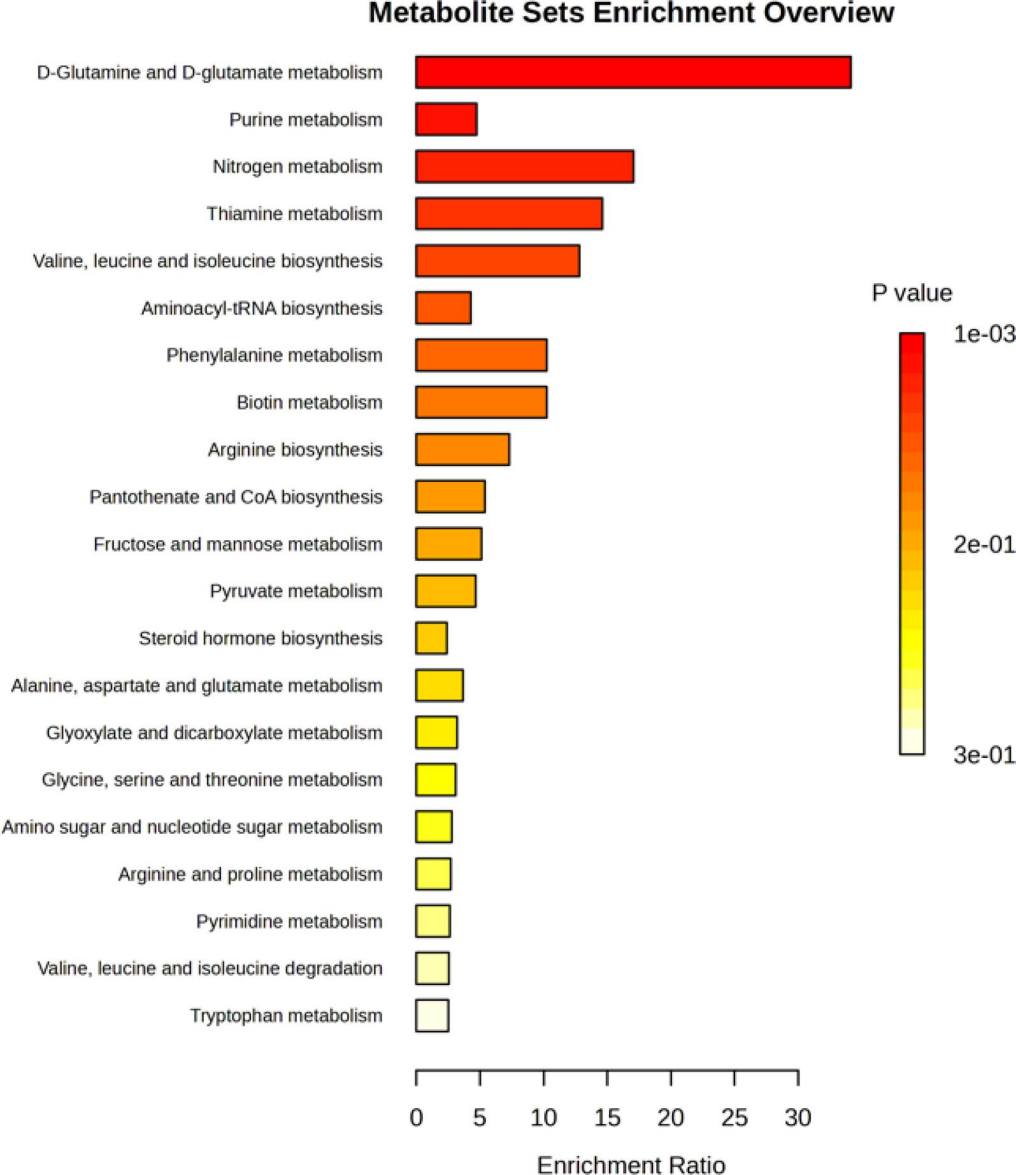
Metabolic pathways identified considering the metabolites differentially expressed in the plasma of acute VTE and controls

Overrepresentation analysis (ORA) was performed, and one-tailed *p* values were provided after adjusting for multiple testing for enrichment analysis (Fig. 5). There were 21 different metabolic pathways evident from the analysis, with the top 5 represented by d-glutamine and d-glutamate metabolism, purine metabolism, nitrogen metabolism, thiamine metabolism, and valine, leucine and isoleucine biosynthesis. Importantly, the metabolic pathway of d-glutamine and d-glutamate was found to be the most relevant pathway and possessed the strongest impact on the acute VTE phenotype (*p* = 0.001, false discovery rate = 0.06).

### RBC metabolome

**Fig. 6:**
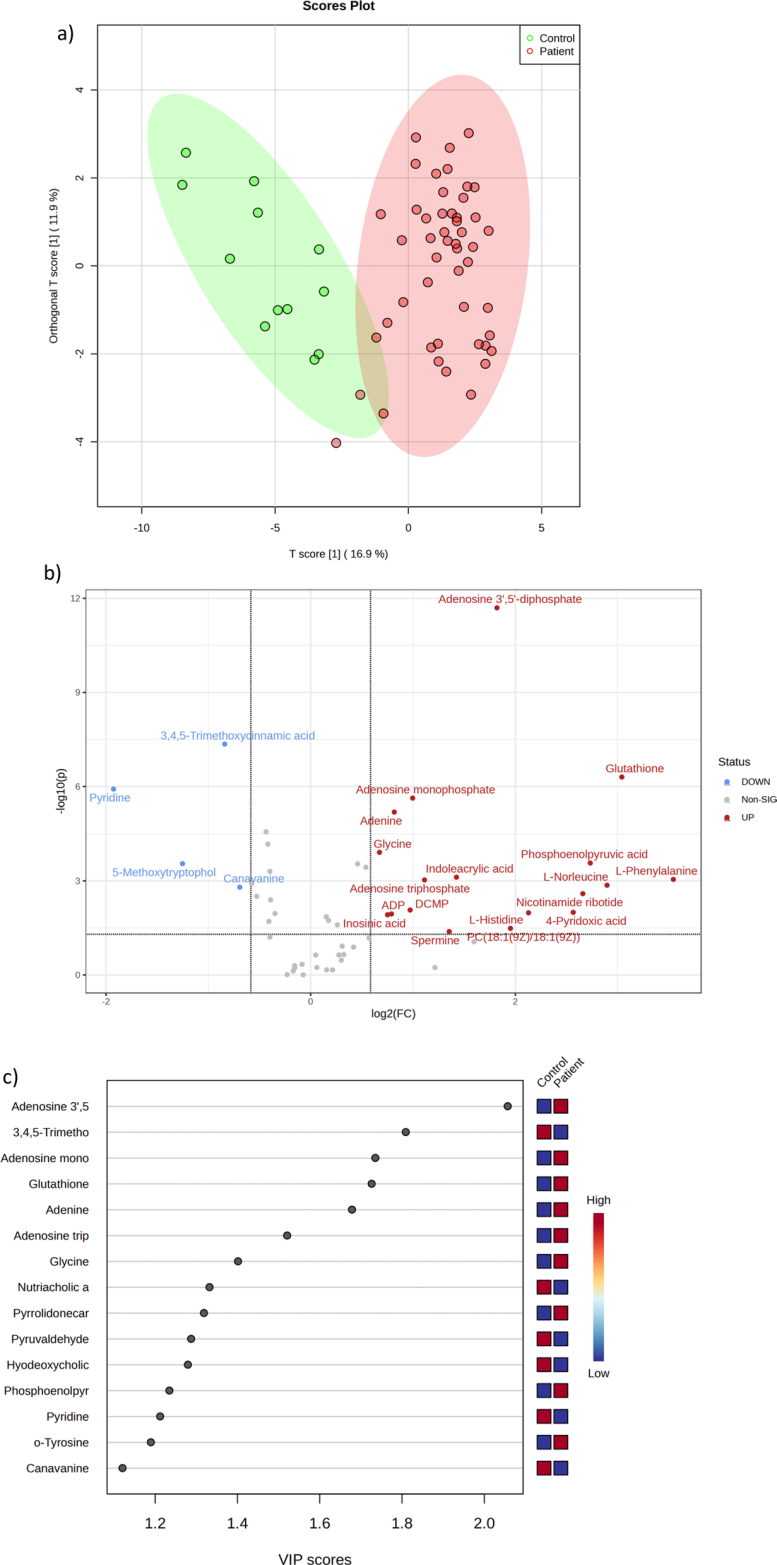

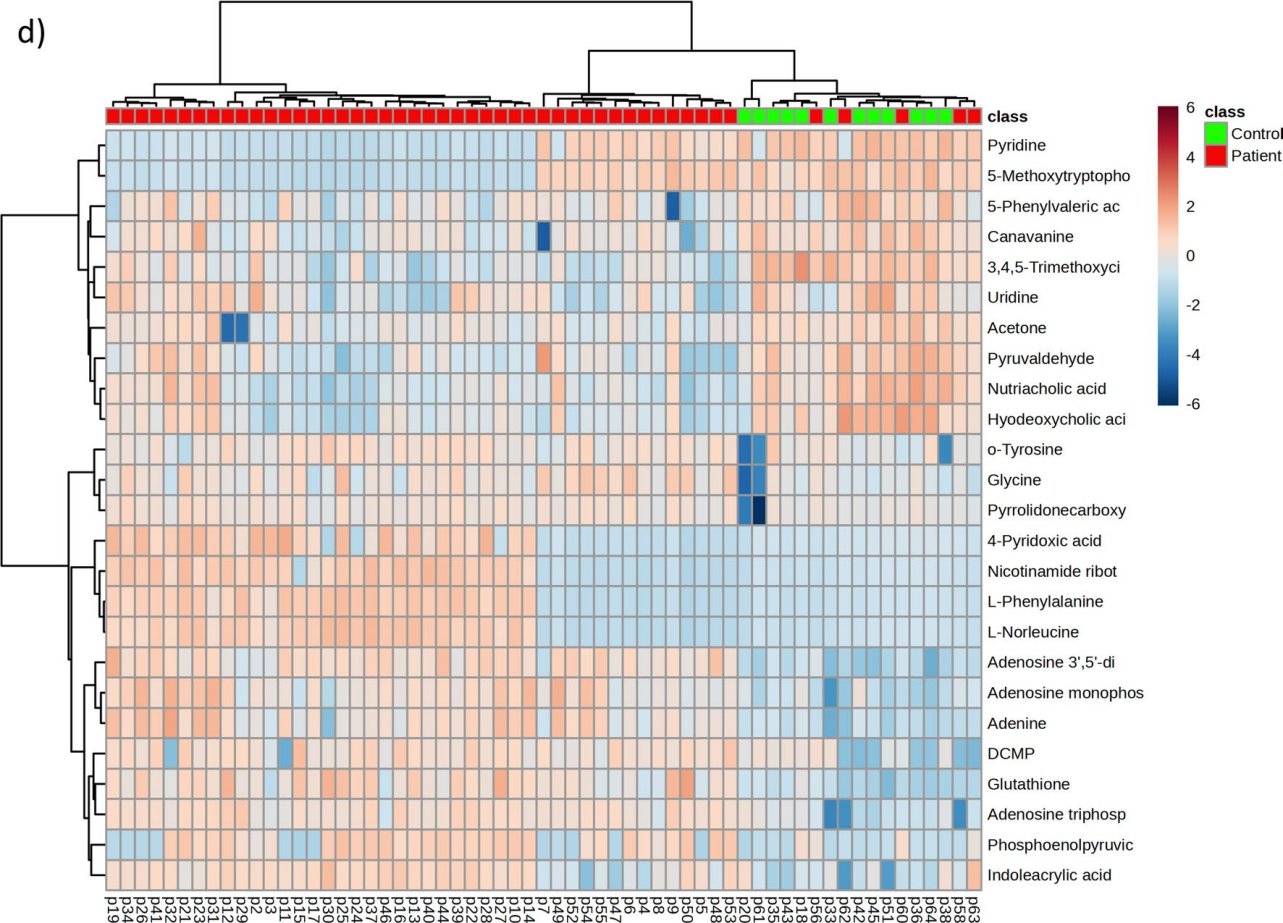
RBCs of the patients with acute VTE exhibit a distinct metabolomic signature. **a** PLS-DA analyses show that the metabolome is able to discriminate between acute VTE patients and control patients, with those metabolites contributing to separation shown in the volcano plot (**b**) and VIP score graph (**c**). **d** Heatmap hierarchical clustering analyses using the 25 metabolites with the lowest *p* value (Wilcoxon test) indicate that there is good clustering between groups

Orthogonal partial least squares-discriminant analysis (PLS-DA) of RBC metabolites, validated by principal component analysis, identified a consistent metabolomic signature of acute VTE (Fig. 6a). The values of the variable impact importance factor (VIP) of the first component in PLS-DA, considered only for metabolites with VIP > 1 and *p* < 0.05, are shown in Fig. 6c. The analysis of the raw data generated a volcano plot (Fig. 6d) and a visual heatmap of the top 25 differentially abundant metabolites (Fig. 6b).

**Fig. 7:**
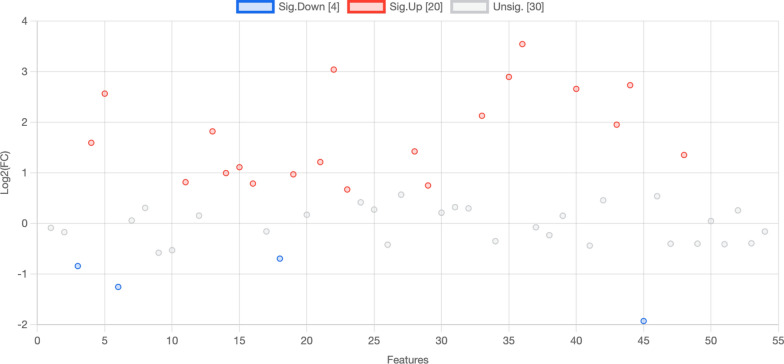
RBCs from acute VTE patients exhibited 24 significantly different metabolites

According to the volcano plot, it can be concluded that the acute VTE group had 20 high metabolites and 4 low metabolites compared to the nonacute VTE group (Fig. 7 and Table 4).

**Table 4:** RBC metabolites significantly different between acute VTE and control patients

	log2(FC^a^)
Adenosine 3′,5′-diphosphate	1.82
3,4,5-Trimethoxycinnamic acid	− 0.84
Glutathione	3.04
Adenosine monophosphate	0.99
Adenine	0.82
Glycine	0.67
Phosphoenolpyruvic acid	2.73
5-Methoxytryptophol	− 1.25
Indoleacrylic acid	1.42
L-Phenylalanine	3.54
Adenosine triphosphate	1.11
L-Norleucine	2.90
Canavanine	− 0.69
Nicotinamide ribotide	2.66
DCMP	0.97
4-Pyridoxic acid	2.57
L-Histidine	2.13
ADP	0.79
Inosinic acid	0.75
PC(18:1(9Z)/18:1(9Z))	1.95
Spermine	1.35

^a^Fold change

**Fig. 8:**
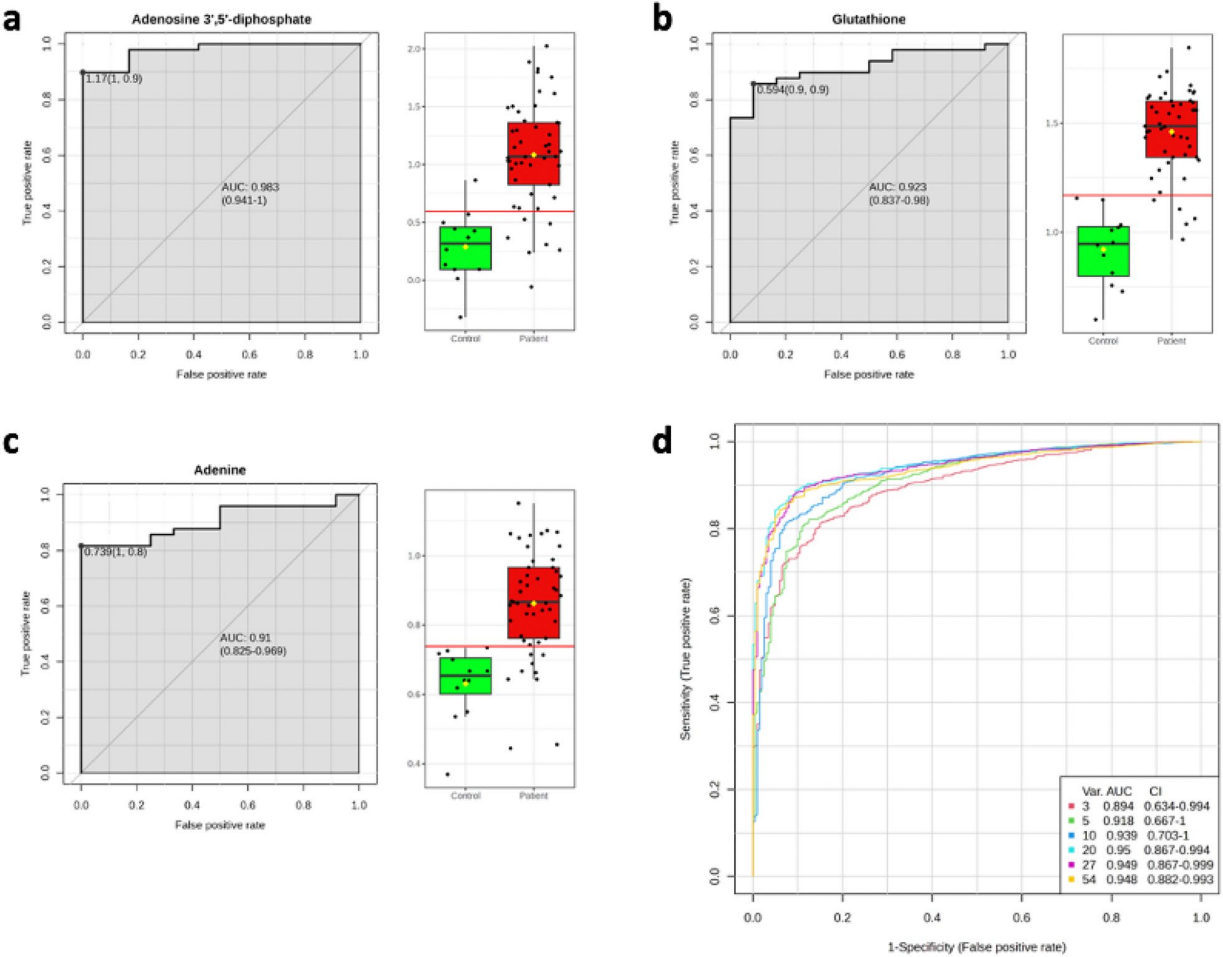
ROC curves of biologically relevant RBC metabolites with AUC > 0.9 values (**a**–**c**) and multivariate ROC analysis (**d**) with confidence intervals of the six models

To determine the diagnostic efficiency of the above 23 potentially differentially abundant metabolites, we generated subject ROC curves and obtained 5 differentially abundant metabolites with a high performance for differentiating acute VTE from nonacute VTE patients. After exclusion of exogenous bioactive metabolites (pyridine and 3,4,5-trimethoxycinnamic acid), there were 3 metabolites with high-performance ROC curves, including adenosine 3′,5′-diphosphate (AUC: 0.983), glutathione (AUC: 0.923), and adenine (AUC: 0.91), whose ROC curves are shown in Fig. 8. The multivariate ROC analysis (Fig. 8d) presents a quasihigh performance for only 3 simultaneous metabolites (AUC: 0.894) and coherently high performance for 5 metabolites (AUC: 0.918 with CI 0.667–1).

**Fig. 9:**
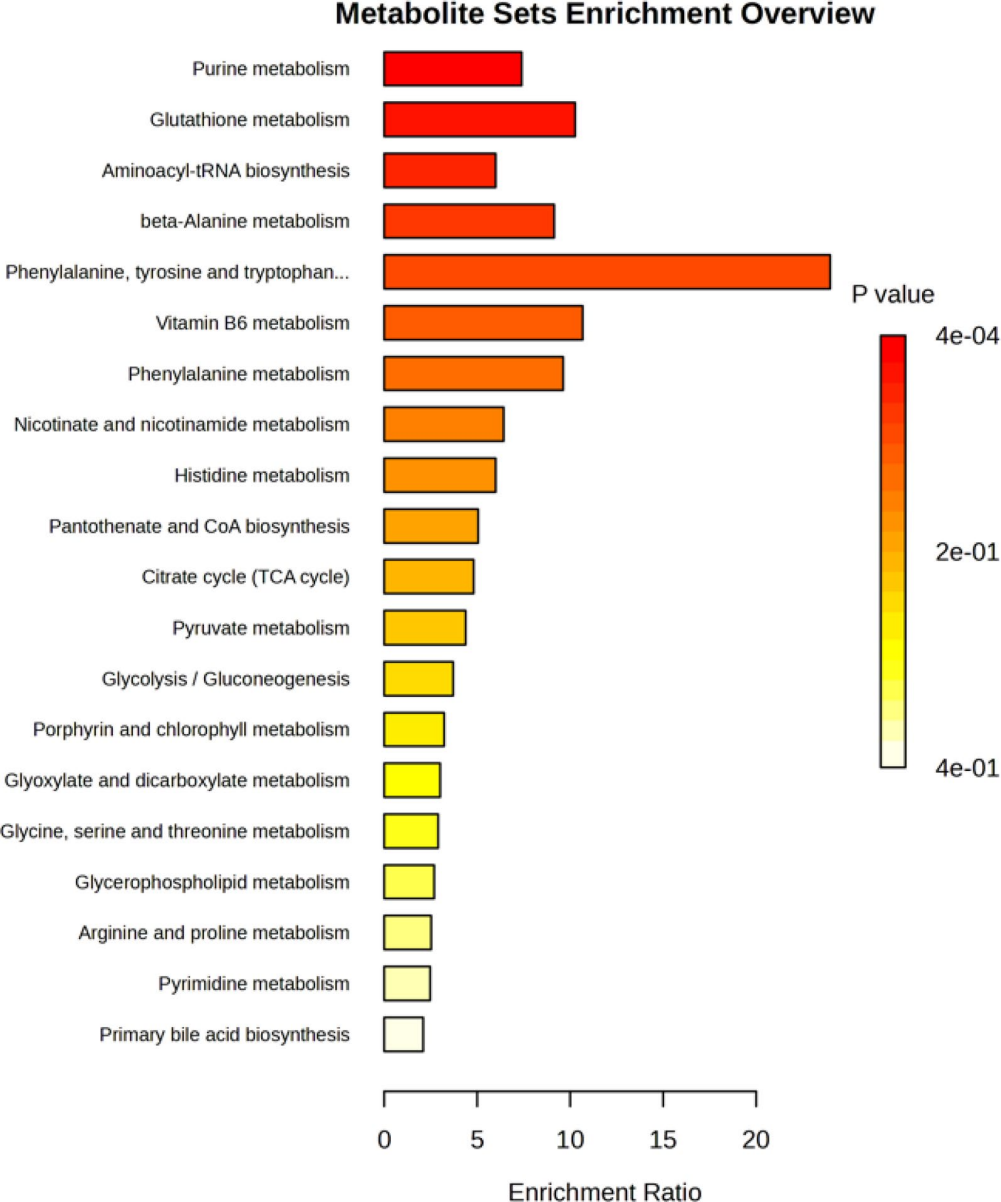
Enrichment analysis of the metabolic pathways identified considering the RBC metabolites differentially expressed between acute VTE and controls

The enrichment analysis of RBC metabolites differentially expressed between acute VTE and non-acute VTE patients resulted in the identification of a total of 20 different pathways (Fig. 9). The metabolic sets most impacting the differences observed were purine metabolism (*p* = 0.000354, false discovery rate = 0.68), glutathione metabolism, aminoacyl-tRNA biosynthesis, and beta-alanine metabolism.

## Discussion

Acute VTE still represents a major diagnostic challenge to physicians worldwide. In this study, 23 plasma metabolites and 24 RBC metabolites were significantly different between acute VTE and nonacute VTE patients. The results of this analysis support the usefulness of high-throughput metabolomic methods to new biomarker findings in cardiovascular diseases [22–24]. The findings of RBCs analysis, in particular, represent novel inaugural data obtained from untargeted LC–MS of patients in the acute clinical phases of VTE. Chromatography is a widely used approach to the discovery of new biomarkers due to its ease of operation, inexpensiveness, selectiveness, reproducibility and low limits of detections [25]. The use of LC–MS in this study reinforces its role in the discovery of new potential biomarkers in VTE. The use of LC–MS in this study reinforces its role in the discovery of new potential biomarkers in VTE.

Plasma metabolome revealed a limited diagnostic performance for acute VTE in this population. Previous untargeted plasma metabolomic assays identified changes in acylcarnitines in post-VTE patients [26, 27], which are involved in the regulation of sugar and lipid metabolism [28]. In our study, the plasma metabolome did not show significant changes in acylcarnitine metabolites, which could be explained by the fact that we analyzed patients in the early acute phases of VTE and the available comparable data were obtained from patients several weeks to months from discharge.

In contrast, the RBC metabolomic signature was clearly discriminatory of acute VTE patients. The role of RBCs in thrombosis extends from interactions with platelets, fibrinogen and coagulation proteins, as they incorporate the initial clot structure [29], modulation of the clot mechanic properties [30], to redox equilibrium and response to extreme conditions such as hypoxia [31]. Nevertheless, new approaches to its metabolism are expected to unshadow some other potential functions [32]. Adenosine 3′,5′-diphosphate (PAP) acts as a sulfuryl group transferring cofactor between compounds with PAP binding sites [33], and its targets in human cells are unknown [34]. PAP is converted to 3′-phosphoadenosine 5′-phosphosulfate (PAPS) by sulfotransferase and then back to PAP, impacting different bioprocesses, such as cell communication, growth and development, and defense [35]. PAP inhibits sulfotransferase even at micromolar concentrations and, consequently, sulfation events on substrate molecules, including heparan sulfate [35]. Heparan sulfate, a ubiquitous protein ligand involved in the regulation of blood coagulation [36], was identified on normal mature human RBCs and is probably involved in heparan sulfate-sensitive adhesion of pathogens such as malarial parasites [37]. In this study, PAP was found to be higher in the RBCs of patients with acute VTE than in those of patients without VTE. The impact of RBC PAP accumulation on sulfation dysregulation and the risk for acute VTE deserves further investigation. The absence of previous data on metabolic profiling of RBCs from acute VTE patients prevents us from comparisons with other studies and increases the cautiousness that we shall use to interpret and generalize them.

All adenine nucleotide metabolites were upregulated in RBCs. ADP is a key intracellular component of the adenosine triphosphate (ATP) cycle that is abundant in RBCs, platelets and other cells. ADP plays an important role in energy metabolism, nucleic acid synthesis, cell signaling and gene expression [38], while its exit from cells results in platelet activation and aggregation [39–41]. Adenosine, derived from adenine, is an inhibitor of platelet activation [39] that is usually low in extracellular environments and increases in response to hypoxia, inflammation, and tissue injury [42]. Adenosine was found to be increased in the vein wall of DVT models and decreased in the serum of severe PE patients [43, 44]. Although we did not evaluate plasma, our findings of increased adenosine in RBCs of acute VTE patients lead us to the hypothesis that during a venous thrombosis event, complex cross-talk between plasma and cells, such as RBCs and endothelial cells, may take place in the regulation of adenosine signaling. The acute VTE patients in our study exhibited significantly higher levels of RBC-derived glutathione, and the oxidative/antioxidative balance shifted towards the oxidative status in venous thromboembolism [45, 46]. Notably, glutathione metabolism can be the potential origin of free radicals interfering with platelet redox status and acting as modulators of platelet function [47]. In the preclinical stages of VTE, inflammation of the endothelium is one of the first phenomena and is responsible for high oxidative stress, with increased glutathione production [48]. Glutathione may thus have a potential role as part of a diagnostic biomarker panel for acute VTE in future studies.

The question of whether arterial and venous thrombosis are just two different clinical manifestations on the continuum of the same disease [49] remains to be answered. Plasma metabolomic studies from patients diagnosed with coronary syndrome identified upregulation of 2-OH-butyric acid, a glutathione presynthesis byproduct under hypoxia [50, 51], in line with our findings of higher glutathione levels in RBCs from acute VTE patients. Purine metabolism has also been associated with unstable angina [52, 53], metabolic syndrome and all-cause mortality [54–56], and venous thrombosis [57, 58]. A similar trend was observed in both plasma and RBCs of our acute VTE, reinforcing possible pathophysiological common pathways. Finally, glutamate and glutamine metabolites, which were higher in the plasma of our acute VTE patients, were previously found to be independently associated with type 2 diabetes [59], stroke and composite cardiovascular disease [60]. There may be reasons to believe in common pathways for arterial and venous acute thrombotic conditions that may be explored by multi-omics.

Despite their promise as early biomarkers for acute VTE, the measurements of new biomarkers are usually costly and dependent on complex laboratory techniques and methods [61]. Clinical metabolomics for biomarker screening of acute VTE should aim for a widely available, fast and inexpensive laboratory analysis that can be measured in real practice. Nucleotides such as adenine can be quantified by LC/MSMS from blood and various tissues and cells [62, 63], with the use of simple extraction and quantification protocols that can be a future solution to daily practice. Glutathione determination is already possible with current clinical laboratory methods and even with simple methods that could allow future minimally invasive monitoring [64]. The daily use of LC‒MS-based metabolomics is not possible in most hospitals. Nevertheless, we believe that this technology, similar to other omics-based technologies, will lead to the discovery of pathophysiological pathways occurring during acute VTE that should enhance hyperacute treatment and the prognosis of these patients. Further translation of these biomarkers to the clinic will certainly require the development of more cost-effective technologies.

Our study has limitations. The groups presented large differences in the size and variance. However, by using controls that are acutely ill patients presenting the same symptoms as acute VTE, we increased our possibilities to identify reliable diagnostic/predictive biomarkers for this pathology. The ROC curves showed high diagnostic accuracy for RBC metabolite data, which confirmed that assumption. The PLS-DA model statistics and heatmap for acute VTE did not result in a perfect distinction between them and nonacute VTE, thus partially limiting the interpretability of the enrichment analysis to the most significant findings, especially in plasma. Similarly, differences in metabolomics profiles due to body mass index could entail changes in the metabolome. Unfortunately, this study does not use a confirmatory cohort, which might be useful in the future for enhancing the robustness of the findings.

## Conclusions

This discovery based metabolomic study using patient plasma and RBCs, clearly suggests that acute VTE presents a specific metabolic signature. The differences in early phases of the disease have potential diagnostic predictive value. Our findings identified a panel of RBCs metabolites that potentially represents a set of diagnostic biomarkers to differentiate patients presenting with an acute VTE episode from other patients. These results should be validated by larger targeted metabolomic studies. Acute venous thrombosis revealed a plasmatic and RBCs metabolomic profile suggestive of disturbances in nucleotide metabolism and of excessive oxidative stress which are common to arterial thrombosis, and point to the existence of some shared pathophysiologic processes.

### Supplementary Information

**Additional file 1: Table S1**. List of the 91 metabolites identified in plasma (without contaminants). **Table S2**. List of the 55 metabolites identified in RBC’s (without contaminants).

## Data Availability

Not applicable.

## Abbreviations

AUC: Area under the curve
DVT: Deep vein thrombosis
ER: Emergency room
ESI: Electrospray ionization
HMBD: Human metabolome database
INSA: National Institute of Health Dr. Ricardo Jorge
LC–MS: Liquid chromatography coupled to mass spectrometry
MS: Mass spectrometry
OPLS-DA: Orthogonal partial-least squares discrimination analysis
ORA: Over representation analysis
PCA: Principle component analysis
PE: Pulmonary embolism
RBCs: Red blood cells
RDW: Red cell distribution width
ROC: Receiver operating characteristics
RPLC: Reversed-phase liquid chromatography
UHPLC: Ultra-high-performance liquid chromatography
UHPLC-ESI-QTOF-MS: Ultra-high-performance liquid chromatography coupled to electrospray ionization and quadrupole time-of-flight mass spectrometry
VTE: Venous thromboembolism
