# Supplementary material for: Acute venous thromboembolism plasma and red blood cell metabolomic profiling reveals potential new early diagnostic biomarkers: observational clinical study
Source: J Transl Med. 2024 Feb 24;22:200. doi: 10.1186/s12967-024-04883-8 (PMC10894498; doi:10.1186/s12967-024-04883-8)
Supplement: Supplementary file 1 — Additional file 1: Table S1. List of the 91 metabolites identified in plasma (without contaminants). Table S2. List of the 55 metabolites identified in RBC’s (without contaminants). [file 12967_2024_4883_MOESM1_ESM.docx]

Table S1 List of the 91 metabolites identified in plasma (without contaminants)

| \| 3223322323231111111111111111111111111231(R)-Lipoic acid \| \| --- \| \| 17a-Estradiol \| \| 1-Methyladenosine \| \| 2,3-Diaminopropionic acid \| \| 2,4-Diaminobutyric acid \| \| 2-Ethyl-2-hydroxybutyric acid \| \| 2-Hydroxy-3-methylbutyric acid \| \| 2-Phenylbutyric acid \| \| 3,4,5-Trimethoxycinnamic acid \| \| 3-Hexenedioic acid \| \| 3-Methylindole \| \| 3-Nitrotyrosine \| \| 4-Aminohippuric acid \| \| 4-Aminophenol \| \| 4-Hydroxyproline \| \| 4-Pyridoxic acid \| \| 5-Hydroxy-L-tryptophan \| \| 5-Methoxytryptophol \| \| Acetone \| \| Adenosine monophosphate \| \| Allantoic acid \| \| All-trans-retinoic acid \| \| Alpha-N-phenylacetyl-L-glutamine \| \| Androstenedione \| \| Aspartyl-lysine \| \| Asymmetric dimethylarginine \| \| Benzoic acid \| \| Biotin \| \| Canavanine \| \| Citramalic acid \| \| Cortisol \| \| Creatinine \| \| Cyclic AMP \| \| Dihydrothymine \| \| DUMP \| \| Elaidic acid \| \| Estradiol \| \| Ethanolamine \| \| Etiocholanolone \| \| Hippuric acid \| \| Homocysteine \| \| Hydroxyindoleacetic acid \| \| Hyodeoxycholic acid \| \| Hypoxanthine \| \| Indoleacrylic acid \| \| Indolelactic acid \| \| Isovalerylcarnitine \| \| L-Acetylcarnitine \| \| L-Arginine \| \| L-Carnitine \| \| L-Cystathionine \| \| Leucinic acid \| \| Levulinic acid \| \| L-Fucose \| \| L-Glutamine \| \| L-Kynurenine \| \| L-Leucine \| \| L-Methionine \| \| L-Norleucine \| \| L-Tryptophan \| \| L-Valine \| \| m-Coumaric acid \| \| Myristic acid \| \| N-Acetyl-L-phenylalanine \| \| Naringenin \| \| Nutriacholic acid \| \| Oleic acid \| \| o-Tyrosine \| \| Pantothenic acid \| \| Paraxanthine \| \| PC(18:1(9Z)/18:1(9Z)) \| \| p-Cresol \| \| Pipecolic acid \| \| Progesterone \| \| Pyridoxal \| \| Pyrrolidonecarboxylic acid \| \| Pyruvaldehyde \| \| Quinaldic acid \| \| Ribothymidine \| \| Saccharopine \| \| Sphinganine \| \| Sphingosine \| \| Stearic acid \| \| Succinylacetone \| \| Thiamine \| \| Tyramine \| \| Urea \| \| Uric acid \| \| Uridine \| \| Xanthosine \| |
| --- | --- | --- | --- | --- | --- | --- | --- | --- | --- | --- | --- | --- | --- | --- | --- | --- | --- | --- | --- | --- | --- | --- | --- | --- | --- | --- | --- | --- | --- | --- | --- | --- | --- | --- | --- | --- | --- | --- | --- | --- | --- | --- | --- | --- | --- | --- | --- | --- | --- | --- | --- | --- | --- | --- | --- | --- | --- | --- | --- | --- | --- | --- | --- | --- | --- | --- | --- | --- | --- | --- | --- | --- | --- | --- | --- | --- | --- | --- | --- | --- | --- | --- | --- | --- | --- | --- | --- | --- | --- | --- |

Table S2 List of the 55 metabolites identified in RBC’s (without contaminants)

| (R)-Lipoic acid |  |
| --- | --- |
| 2-Phenylbutyric acid |  |
| 3,4,5-Trimethoxycinnamic acid |  |
| 3-Phenylbutyric acid |  |
| 4-Pyridoxic acid |  |
| 5-Methoxytryptophol |  |
| 5-Methylcytidine |  |
| 5'-Methylthioadenosine |  |
| 5-Phenylvaleric acid |  |
| Acetone |  |
| Adenine |  |
| Adenosine |  |
| Adenosine 3',5'-diphosphate |  |
| Adenosine monophosphate |  |
| Adenosine triphosphate |  |
| ADP |  |
| Androstenedione |  |
| Canavanine |  |
| DCMP |  |
| Deoxyguanosine |  |
| Diacetyl |  |
| Ethanolamine |  |
| Glutathione |  |
| Glycine |  |
| Glycylproline |  |
| Guanosine 5'-diphosphate |  |
| Guanosine monophosphate |  |
| Hyodeoxycholic acid |  |
| Hypoxanthine |  |
| Indoleacrylic acid |  |
| Inosinic acid |  |
| Isovalerylcarnitine |  |
| L-Acetylcarnitine |  |
| L-Carnitine |  |
| L-Histidine |  |
| Linoleic acid |  |
| L-Norleucine |  |
| L-Phenylalanine |  |
| L-Tryptophan |  |
| m-Coumaric acid |  |
| Nicotinamide ribotide |  |
| Nutriacholic acid |  |
| o-Tyrosine |  |
| PC(18:1(9Z)/18:1(9Z)) |  |
| Phosphoenolpyruvic acid |  |
| Pyridine |  |
| Pyrrolidonecarboxylic acid |  |
| Pyruvaldehyde |  |
| Spermine |  |
| Sphinganine |  |
| Succinylacetone |  |
| Tyramine |  |
| Uric acid |  |
| Uridine |  |
| Uridine 5'-monophosphate |  |
